# Long-acting dihydropyridine calcium-channel blockers and sympathetic nervous system activity in hypertension: A literature review comparing amlodipine and nifedipine GITS

**DOI:** 10.3109/08037051.2012.690615

**Published:** 2012-07-05

**Authors:** Corey B. Toal, Peter A. Meredith, Henry L. Elliott

**Affiliations:** 1 Department of Pharmacology, University of Toronto, Toronto, Canada; 2 University of Glasgow, Glasgow, UK; 3 University of Strathclyde, Glasgow, UK

**Keywords:** Amlodipine, blood pressure, catecholamines, extended release, GITS, nifedipine, norepinephrine, sympathetic activation, sympathetic nerve activity

## Abstract

Calcium-channel blockers (CCBs) constitute a diverse group of compounds but are often referred to as a single homogeneous class of drug and the clinical responses indiscriminately summarized. Even within the dihydropyridine subgroup, there are significant differences in formulations, pharmacokinetics, durations of action and their effects on blood pressure, heart rate, end organs and the sympathetic nervous system. Amlodipine and nifedipine in the gastrointestinal therapeutic system (GITS) formulation are the most studied of the once-daily CCBs. Amlodipine has an inherently long pharma-cokinetic half-life, whereas, in contrast, nifedipine has an inherently short half-life but in the GITS formulation the sophisticated delivery system allows for once-daily dosing. This article is derived from a systematic review of the published literature in hypertensive patients. The following search terms in three main databases (MEDLINE, Embase, Science Citation Index) from 1990 to 2011 were utilized: amlodipine, nifedipine, sympathetic nervous system, sympathetic response, sympathetic nerve activity, noradrenaline, norepinephrine and heart rate. More than 1500 articles were then screened to derive the relevant analysis. As markers of sympathetic nervous system activation, studies of plasma norepinephrine concentrations, power spectral analysis, muscle sympathetic nerve activity and norepinephrine spillover were reviewed. Overall, each drug lowered blood pressure in hypertensive patients in association with only small changes in heart rate (i.e. < 1 beat/min). Plasma norepinephrine concentrations, as the most widely reported marker of sympathetic nervous system activity, showed greater increases in patients treated with amlodipine than with nifedipine GITS. The evidence indicates that both these once-daily dihydropyridine CCBs lower blood pressure effectively with minimal effects on heart rate. There are small differences between the drugs in the extent to which each activates the sympathetic nervous system with an overall non-significant trend in favour of nifedipine GITS.

## Introduction

Calcium-channel blockers (CCBs), comprise three distinct subgroups: benzothiazepines (e.g. diltiazem), dihydropyridines (e.g. amlodipine, nifedipine) and phenylalkylamines (e.g. verapamil). Despite this diversity, they are often referred to as a single, homogeneous class of pharmacological agents. Furthermore, even within the dihydropyridine group, there are numerous drugs and formulations (e.g. nifedipine capsules, retard, gastrointestinal therapeutic system: GITS) with different pharmacokinetic profiles, clinical uses and responses, and different dosing requirements.

Despite these various pharmacokinetic differences, arterial vasodilatation is the fundamental response to calcium-channel blockade with a dihy-dropyridine CCB. Peripheral arterial vasodilatation leads to a reduction in blood pressure and coronary artery vasodilatation leads to increased blood flow to the myocardium. However, it has long been known that potent arterial vasodilators evoke a barorecep-tor-mediated reflex increase in heart rate that is mediated via the sympathetic nervous system. This holds for both arterial vasodilators, such as nifedipine and hydralazine (1), and for mixed arterio-venous dilators such as nitroglycerin (2). Thus, the positive consequences of arterial vasodilatation may be compromised by activation of the sympathetic nervous system and an increase in heart rate.

Insights into the balance between these positive and negative effects became apparent in the results of the early studies with nifedipine in its immediate release formulation: two clinical outcome studies indicated that in patients with unstable angina (3) and post-myocardial infarction (4), the administration of nifedipine, as a potent arterial vasodilator, did not lead to a clear reduction in morbidity and mortality. At the time, this appeared counter-intuitive because coronary vasodilatation in both these conditions would be expected to increase oxygen delivery to the myocardium and benefit the patients, as would the reduction in cardiac work through the reduction in afterload. However, in hindsight, reflex sympathetic activation, catecholamine release and increased heart rate would be likely to have offset the expected beneficial effect.

In a later review, Grossman & Messerli (5) suggested that rapid-onset, short-acting dihydropyridine CCBs evoked sympathetic activation, whether administered acutely or over several weeks. In contrast, long-acting dihydropyridine CCBs did not evoke the same response. However, this is an over-simplification: for example, the once-daily ER formulation of the dihydropyridine drug felodipine has been shown to elevate plasma catecholamines (a marker of sympathetic activation) and result in less left ventricular regression in hypertensive patients compared with either enalapril or nifedipine GITS (6,7).

Despite obvious differences between drugs, between classes or subgroups or formulations, CCBs are often indiscriminately grouped together. They are often summarized as a single entity in reviews of outcome trials and when reviewed by formulary committees and by funding organizations. This raises obvious questions about the most appropriate method of considering the interchangeability of different CCBs and, for this reason, we decided to conduct a detailed review of the literature on two of the most commonly used dihydropyridine CCBs, amlodipine and nifedipine GITS, with specific regard to their effects on sympathetic activation.

## Methods

### Literature review

The MEDLINE (Pubmed), Embase, Derwent Drug File, Biosis and Science Citation Index databases were searched for articles published between 1990 and April 2011 on amlodipine and nifedipine using the terms *sympathetic nervous system, sympathetic response, sympathetic nerve activity, noradrenaline, norepinephrine, heart rate, hypertension*. We included only articles published in the English language. If a study was published in more than one journal, efforts were made only to include the data once from whichever article was most complete in study details and data. The primary focus was on full manuscript publications and not abstracts. However, if an abstract was published but a full paper was not subsequently found, the abstract was used if there were data on number of patients, dose of drug, duration of treatment and relevant measurement values. More than 1500 articles were screened and only those in which treatment lasted for at least 1 week were included in the analysis.

### Indices of sympathetic nervous system activity

The following measurements of sympathetic nervous system activity/sympathetic activation were evaluated:

(1) plasma norepinephrine (noradrenaline) concentrations;
(2) muscle sympathetic nerve activity recordings;
(3) power spectral analyses of low-frequency and high-frequency activity.

All plasma concentration values for norepineph-rine were converted to pg/ml and changes were calculated as percentage (%) values. Sympathetic activation values were included for patients at rest as distinct from those stimulated by mental stress, handgrip, standing or cold pressor tests.

### Background details

Generally, data were reported for patients in the supine or sitting position. For the blood pressure measurements, office- or clinic-based values are incorporated and average daytime values if ambulatory blood pressure readings were used. If the final blood pressure, heart rate or other measurement was not given as an absolute value but as a change from baseline, the end of measurement value was calculated by simply adding the mean change value to the initial/baseline value. To make allowances for different baseline values, different study designs, different methodologies etc., percentage changes from baseline to the end time point have been calculated. If measurements were made at multiple time points within one published study, the longest duration of treatment was chosen and if multiple doses were reported, or dose titration occurred, the final dose of drug or the most-used dose is reported.

### Statistics

Only one study permitted a direct statistical comparison (8). It was adjudged that formal statistical testing was not otherwise appropriate because of wide variability in the results and because of significant differences in methodologies, study characteristics and relatively small study numbers. Thus, summary statistics (means, standard error and percentage change) were used to make comparisons between the drugs.

## Results

### Plasma norepinephrine

Measurement of plasma norepinephrine concentrations was the most commonly reported index of sympathetic activity and activation. Twenty-three (23) studies were identified for amlodipine and 14 (14) for nifedipine GITS. For amlodipine, 698 patients were evaluated with an overall mean age of 56 years (Table I). Corresponding mean values for nifedipine GITS were 291 patients and 57 years (Table II). There was considerable variability in the plasma norepinephrine results in that the changes from baseline ranged from—21.4% to 55.6% with amlodipine and from—3.1% to 58.9% with nifedipine GITS.

**Table I:** Studies on amlodipine reporting plasma norepinephrine.

Reference^a^	Year	*n*	Mean age (years)	Dose (mg)	Time interval (weeks)	%Δ in SBP	%Δ in DBP	%Δ in HR	%Δ in NE
Lopez et al. (20)	1990	12	61	2.5–10	4	–8.1	–8.9		35.1
Donati et al. (21)	1992	10	47	5	8	–11.3	–9.3	–5.6	–8.7
Leenen and Fourney (22)	1996	17	55	10	26	–13.3	–12.7		–11.1
Sasaguri et al. (23)	1997	8		5	1	–13.2	–8.8	1.2	3.0
Hamada et al. (24)	1998	16	60	5	4	–10.5	–7.2	–1.4	–21.4
de Champlain et al (8)	1998	22	55	10	6	–10.0	–12.6	8.0	55.6
Sakata et al. (25)	1999	24	63	10	12	–17.7	–20.2	0.0	18.9
Malamani et al. (26)	1999	60		10	12				41.7
Spence et al. (27)	2000	24	47	10	4	–7.9	–8.9	4.1	27.7
Fogari et al. (28)	2000	15	55	10	24	–11.9	–13.7	1.4	34.9
Lefrandt et al. (29)	2001	145	51	5	8	–9.8	–9.0	1.5	23.2
Struck et al. (30)	2002	18	56	5	1	–9.1	–5.3	6.0	33.7
Eguchi et al. (31)	2002	46	69	10	8	–17.3	–10.9	–2.9	23.8
Binggeli et al. (32)	2002	14	58	5	8	–9.7	–9.6	–4.6	47.1
Ohbayashi et al. (33)	2003	37	68	5	26	–1.4	–1.3	0.0	13.0
Malacco et al. (34)	2004	46	57	10	12	–9.8	–12.6	2.7	15.2
Karas et al. (35)	2005	22	57	10	8	–14.3	–12.0		48.8
Leenen et al. (36)	2006	29	41	5	8	–4.4	–15.1		19.3
Leenen et al. (36)	2006	37	67	5	8	–0.7	–11.4		7.4
Ruzicka et al. (37)	2007	10	42	5	6	–4.6	–4.4	–2.7	18.6
de Champlain et al. (38)	2007	23	57	10	8	–12.8	–12.4	1.4	38.2
Larochelle et al. (39)	2008	42	58	10	8	–12.3	–11.6		38.1
Sanjuliani et al. (40)	2002	21	47	10	26	–15.2			–3.6
Total		698							
Mean			55.8						
Mean % change						–10.2	–10.4	0.6	21.7
SE						0.94	0.83	0.78	4.27

SBP, systolic blood pressure; DBP, diastolic blood pressure; HR, heart rate; NE, norepinephrine; %Δ, percentage change.

a Each reference is an independent study published reporting on the relevant parameters indicated with % changes calculated on the group means.

**Table II:** Studies on nifedipine gastrointestinal therapeutic system reporting plasma norepinephrine.

Reference^a^	Year	*n*	Mean age (years)	Dose (mg)	Time interval (weeks)	%Δ in SBP	%Δ in DBP	%Δ in HR	%Δ in NE
Frohlich et al. (41)	1991	10	52	65	8	–12.6	–10.9	5.7	–3.1
Phillips et al. (42)	1992	16	56	30–150	52	–28.0	–27.0	–3.7	–1.5
Halperin et al. (43)	1993	12	53	30–90	4	–8.3	–11.1	–2.4	16.8
DeQuattro and Lee (44)	1997	23	66	30–120	12	–12.2	–10.2		40.0
de Champlain et al. (8)	1998	22	51	30–60	6	–8.6	–10.9	0.0	0.0
James et al. (45)	1999	14	70	30–60	6	–12.3	–12.8	–3.0	–0.5
Pellizer et al. (46)	2001	8	57	60	6	–7.5	–8.7	–1.3	58.9
Diamond et al. (47)	2001	15	46	30–120	26	–17.4	–18.2	1.2	0.0
Leenen et al. (7)	2002	17	55	30	30	–9.9	–10.0		57.0
Fogari et al. (48)	2003	30	55	60	48	–13.2	–14.9	0.0	26.0
Ruzicka et al. (49)	2004	10	45	20	4	–0.8	–1.1	2.9	4.3
Ruzicka et al. (49)	2004	8	67	20	4	–6.8	–8.0	7.7	6.6
Fogari et al. (50)	2005	62	59	60	12	–9.8	–12.7	2.7	19.9
Brown and Toal (51)	2007	44	63	30	2	–6.0		–1.4	14.3
Total		291							
Mean			56.8						
Mean % change						–10.9	–12.0	0.7	17.1
SE						1.68	1.60	0.94	5.68

SBP, systolic blood pressure; DBP, diastolic blood pressure; HR, heart rate; NE, norepinephrine; %Δ, percentage change.

a Each reference is an independent study published reporting on the relevant parameters indicated with %changes calculated on the group means.

The changes in blood pressure (BP) and heart rate were similar with the two drugs. With amlodipine, systolic blood pressure (SBP) decreased by 10.2±0.9%, diastolic blood pressure (DBP) by 10.4±0.8% and heart rate increased by 0.6±0.8% (Table I). With nifedipine GITS, the respective changes were 10.9±1.7%, 12.0±1.6% and 0.7±0.9% (Table II).

**Figure 1:**
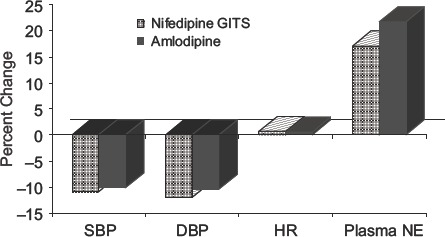
The effects of amlodipine and nifedipine gastrointestinal therapeutic system (GITS) on systolic blood pressure (SBP), diastolic blood pressure (DBP), heart rate (HR) and plasma norepinephrine (NE) when the drug is given over weeks of treatment. This figure is based on the mean percentage changes from Tables I and II for all the studies cited.

In summary, plasma norepinephrine increased by 21.7±4.3% after amlodipine and by 17.1±5.7% after nifedipine GITS (Figure 1).

### Muscle sympathetic nerve activity

Measurement of muscle (peroneal nerve) sympathetic activity was the second most commonly used method and was employed in five amlodipine studies and in one study with nifedipine GITS. In the amlodipine studies, a total of 70 patients with an average age of 52 years were evaluated (Table III). For nifedipine GITS, the single small study split the 18 patients into older and younger groups for evaluation but the overall mean age was similar at 56 years (Table III).

**Table III:** Studies on amlodipine and nifedipine gastrointestinal therapeutic system (GITS) reporting muscle sympathetic nerve activity.

Reference^a^	Year	*n*	Mean age (years)	Dose (mg)	Time interval (weeks)	%Δ in SBP	%Δ in DBP	%Δ in HR	%Δ in MSA
Amlodipine									
Calhoun (52)	1997	10	47	10	4	–9.6	–7.6	2.9	40.0
Binggeli et al. (32)	2002	14	58	5	8	–9.7	–9.6	–4.6	6.1
Struck et al. (30)	2002	18	56	5	1	–9.1			32.1
Ruzicka et al. (37)	2007	10	42	5	6	–4.6	–4.4	–2.7	–3.9
Dodt et al. (53)	2000	18	56	5	1	–9.1	–5.3	6.0	32.6
Total		70							
Mean			51.8						
Mean % change						–8.4	–6.7	0.4	21.4
SE						0.97	1.04	2.19	8.54
Nifedipine GITS									
Ruzicka et al. (49)	2004	10	45	20	4	–0.76	–1.012	2.94	4.88
Ruzicka et al. (49)	2004	8	67	20	4	–6.85	–8.05	3.08	8.51
Total		18							
Mean			56						
Mean % change						–3.81	–4.53	3.01	6.69
SE						3.04	3.52	0.07	1.82

SBP, systolic blood pressure; DBP, diastolic blood pressure; HR, heart rate; MSA, muscle sympathetic nerve activity; %Δ, percentage change.

a Each reference is an independent study published reporting on the relevant parameters indicated with % changes calculated on the group means.

The changes in SBP, DBP and heart rate were 8.4±1.0%, 6.7±1.0% and 0.4±2.2%, respectively, in the amlodipine studies and the corresponding values were 3.8±3.0%, 4.5±3.5% and 3.0±0.1% in the nifedipine studies.

**Figure 2:**
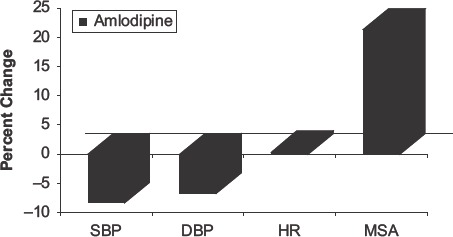
The effects of amlodipine on systolic blood pressure (SBP), diastolic blood pressure (DBP), heart rate (HR) and muscle sympathetic nerve activity (MSA) when the drug is given over weeks of treatment. This figure is based on the mean percentage changes from Table III for all the studies cited.

In summary, the increase in muscle sympathetic nerve activity was 21.4±8.5% for amlodipine and 6.7±1.8% for nifedipine GITS (Figure 2).

### Power spectral analysis

This methodology involves continuous measurement of the ECG to tease out R–R interval variations using fast Fourier transformation and autoregressive algorithms (9). The low-frequency component of the power spectrum is an indicator of sympathetic nerve activity to the heart and high-frequency activity is a measure of parasympathetic activity. Ten studies on amlodipine in hypertensive patients looked at these measures. The average age of the 180 patients studied was 54 years (Table IV). There were no such studies with nifedipine GITS.

**Table IV:** Studies on amlodipine reporting power spectral analysis.

Reference^a^	Year	*n*	Mean age (years)	Dose (mg)	Time interval (weeks)	%Δ in SBP	%Δ in DBP	%Δ in HR	%Δ in LF	%Δ in HF	%Δ in LF/HF
Minami et al. (54)	1998	20	63	5	4	–7.43	–4.55	1.37	–1.38	–5.60	10.29
Hamada et al. (24)	1998	16	60	5	4	–10.49	–7.23	–1.45	–3.52	16.67	–12.50
Lucini et al. (55)	1999	19	54	5	8	–12.73	–11.58	4.29	–9.84		
Siche et al. (56)	2001	18		8	8	–11.41			–62.00	–33.33	
Sahin et al. (57)	2004	20	48	10	4	–28.74	–14.00		22.22	35.48	–10.34
Karas et al. (35)	2005	22	57	10	8	–14.29	–12.00		25.00	11.11	73.33
Bilge et al. (58)	2005	14	46	10	13	–11.11	–11.70	–1.22	–1.48	–2.17	0.76
Bilge et al. (58)	2005	14	46	10	26	–13.19	–13.83	–3.66	–2.31	–2.82	0.00
Linqvist et al. (59)	2007	14	59	10	6	–12.94	–9.89	6.06	–7.25	16.13	–26.47
de Champlain et al. (38)	2007	23	57	10	8	–12.84	–12.37	1.41	7.41	20.00	60.00
Total		180									
Mean			54.4								
Mean % change						–13.52	–10.79	0.97	–3.31	6.16	11.88
SE						1.80	1.04	1.28	7.51	6.60	12.62

SBP, systolic blood pressure; DBP, diastolic blood pressure; HR, heart rate; LF, low frequency; HF, high frequency; LF/HF, ratio of low frequency to high frequency; %Δ, percentage change.

a Each reference is an independent study published reporting on the relevant parameters indicated with % changes calculated on the group means.

**Figure 3:**
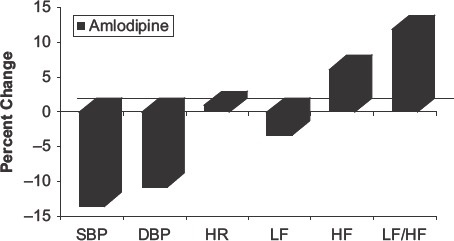
The effects of amlodipine on systolic blood pressure (SBP), diastolic blood pressure (DBP), heart rate (HR), low frequency (LF), high frequency (HF) and the ratio of low to high frequency (LF/HF) of the power spectrum when the drug is given over weeks of treatment. This figure is based on the mean percentage changes from Table IV for all the studies cited.

In the amlodipine-treated patients, the decreases in SBP, DBP and heart rate were 13.5±1.8%, 10.8±1.0% and 1.0±1.3% (Table IV). The low-frequency component of the power spectrum decreased by 3.3±7.5%, the high-frequency component increased by 6.2±6.6% and the ratio of low to high frequency increased by 11.9±12.6% (Figure 3).

## Discussion

The activity of the sympathetic nervous system is essential for the moment-to-moment regulation of the cardiovascular system but “overactivity” has been implicated in both the genesis of, and the complications of, cardiovascular disease (10,11). With regard to treatment effects, specifically with dihydropyridine CCBs, it has been reported that the short-acting and long-acting drugs have distinctly different effects on the sympathetic nervous system (5). This conclusion was in complete accord with the findings in the seminal studies of Kleinbloesem et al. (12,13), which demonstrated that the “rate of drug delivery” was critical for determining the rate of onset of vasodilatation and hence the reflex effects expressed via the sympathetic nervous system. Thus, formulations of dihydropyridine CCBs, which result in a steep rise in plasma drug concentrations, have been shown to activate the sympathetic nervous system, e.g. nife-dipine capsule, nifedipine retard or felodipine (7,8,12–14). In summary, sympathetic activation is typically seen with short-acting dihydropyridine CCBs but the fundamental factor is rapid-onset vasodilatation.

In the earlier review by Grossman & Messerli (5), long-acting agents were indiscriminately grouped together. This present overview compares the evidence derived in studies of the two established long-acting, once-daily dihydropyridine CCBs, which have the greatest volume of clinical outcome evidence: amlodipine an agent with an intrinsically long pharmacokinetic elimination half-life and nifedipine GITS, a high-tech osmotic delivery system, which confers extended release characteristics (15).

Unfortunately, there is only one study that directly compares amlodipine and nifedipine GITS: the conclusion of this single study was that chronic treatment with amlodipine was associated with sympathetic activation, whereas no such activation occurred with nifedipine GITS. This finding is consistent with the overall trend in this present analysis albeit there was no statistical significance. However, the changes in baseline for plasma norepinephrine do not appear to be directly related to the blood pressure lowering effect, since the blood pressure decreases were very similar between the two drugs with, if anything, a marginally greater decrease with nifedipine GITS. Furthermore, the heart rate changes were comparable with the two drugs at approximately 0.6 beats/min.

Thus, the main finding of this overview is that amlodipine (despite its “positive” profile in clinical outcome studies) caused a small but significant activation of the sympathetic nervous system, as assessed by multiple markers. It is also noteworthy that, for measurements of plasma norepinephrine and assessment of muscle sympathetic activation, the percentage increases are coincidentally almost identical (i.e. 21.7% and 21.4%, respectively). In turn, the ratio of low to high frequency from the power spectral analysis suggests that the sympathetic activation component overall is greater than the parasympathetic component after amlodipine administration. Therefore, there is consistency in three different surrogate measures for sympathetic activation, suggesting that amlodipine increases activity of the sympathetic nervous system in hypertensive patients.

A detailed explanation for the apparent differential effects of amlodipine and nifedipine GITS on the sympathetic nervous system is not readily apparent. However, in studies of spontaneously hypertensive rats (SHR), Huang & Leenen (16) concluded that, even during chronic amlodipine administration, there was a balance between peripheral effects and central effects, whereby the plasma concentration of drug might influence the activation of the sympathetic nervous system. Similar results were obtained in a study with nifedipine in SHR (17). However, a slow peripheral intravenous infusion of nifedipine in SHR resulted in a sympatho-inhibitory response–decrease in blood pressure, renal sympathetic nerve activity and heart rate. The GITS osmotic delivery system with nife-dipine may be thought to mimic more closely a slow infusion of drug (relative to other formulations e.g. capsules, Retard) and explain to some degree the more neutral effects seen with nifedipine GITS compared with previous formulations. Taken together, these studies with amlodipine and nifedipine GITS suggest that BP reduction and the effects on the sympathetic nervous system reflect a dynamic equilibrium between central and peripheral effects, which, in turn, are dependent on peripheral (plasma) and central (central nervous system) concentrations of drug.

Notwithstanding the above observations and discussion, the results of this overview must be taken in proper context. The data are compiled from studies spanning some 20 years with patients of varying durations of hypertension, severity of disease, baseline blood pressures, conditions for sampling or measurements, time of day, body posture, dose of drug, age of patient etc. Moreover, no formal statistical analyses were conducted because of varying methodologies and study characteristics, and widely varying results. All of these are potentially complicating and confounding factors when trying to compare drugs. Ideally, a study comparing the two drugs in a head-to-head manner with proper randomization and assessment should provide more robust data. To date, only one such study was found, that by de Cham-plain et al. (8). In that study, although amlodipine did not result in a transient rise in plasma norepi-nephrine after either acute or chronic dosing, administration for 6 weeks was reported to cause a 50% increase in the overall basal concentration of plasma norepinephrine. This was not observed with nife-dipine GITS.

Chronic activation of the sympathetic nervous system has been implicated in the pathogenesis of hypertension and its cardiovascular complications. However, both amlodipine and nifedipine GITS have positive outcome data in the treatment of hypertensive patients (i.e. ALLHAT, INSIGHT respectively (18,19)). This suggests that there is a balance between the benefits of lowering blood pressure and the potentially adverse consequences of sympathetic activation. In practice, therefore, effective BP reduction may be more important than modest sympathetic activation. There are additional considerations, the most obvious of which is that many patients require multiple drugs to manage their blood pressure and the interaction of these other drugs in conjunction with the CCBs may have some counterbalancing effect. Overall, despite the view fostered by the major hypertension treatment guidelines, it is apparent that the dihydropyri-dine CCBs cannot be considered a homogenous group of compounds. Furthermore, even two long-acting once-a-day drugs like amlodipine and nife-dipine GITS, with similar clinical profiles, may have both qualitatively and quantitatively different effects on the sympathetic nervous system.
